# Chemical Profile, Antioxidant and Antibacterial Activities of *Achillea moschata* Wulfen, an Endemic Species from the Alps

**DOI:** 10.3390/molecules21070830

**Published:** 2016-06-25

**Authors:** Sara Vitalini, Moira Madeo, Aldo Tava, Marcello Iriti, Lisa Vallone, Pinarosa Avato, Clementina Elvezia Cocuzza, Paolo Simonetti, Maria Pia Argentieri

**Affiliations:** 1 Dipartimento di Scienze Agrarie ed Ambientali, Università degli Studi di Milano, via G. Celoria 2, 20133 Milano, Italy; marcello.iriti@unimi.it; 2 Dipartimento di Medicina e Chirurgia, Università degli Studi di Milano-Bicocca, Via Cadore 48, 20900 Monza, MB, Italy; moira.madeo@gmail.com (M.M.); clementina.cocuzza@unimib.it (C.E.C.); 3 Consiglio per la Ricerca in Agricoltura e l’Analisi dell’Economia Agraria—Centro di Ricerca per le Produzioni Foraggere e Lattiero Casearie (CREA-FLC), viale Piacenza 29, 26900 Lodi, Italy; aldo.tava@crea.gov.it; 4 Dipartimento di Scienze Veterinarie per la Salute, la Produzione Animale e la Sicurezza Alimentare Università degli Studi di Milano, via Grasselli 7, 20137 Milano, Italy; lisa.vallone@unimi.it; 5 Dipartimento di Farmacia-Scienze del Farmaco, Università degli Studi di Bari “Aldo Moro”, via Orabona 4, 70125 Bari, Italy; pinarosa.avato@uniba.it (P.A.); mariapia.argentieri@uniba.it (M.P.A.); 6 Dipartimento di Scienze per gli Alimenti, la Nutrizione e l’Ambiente, Università degli Studi di Milano, via G. Celoria 2, 20133 Milano, Italy; paolo.simonetti@unimi.it

**Keywords:** *Achillea moschata*, alpine medicinal plants, alpine ethnobotany, phenolic acids, flavonoids, essential oil, antimicrobial activity, antiradical activity

## Abstract

Aerial parts of *Achillea moschata* Wulfen (Asteraceae) growing wild in the Italian Rhaetian Alps were investigated to describe, for the first time, their phenolic content, as well as to characterize the essential oil. Inspection of the metabolic profile combining HPLC-DAD and ESI-MS/MS data showed that the methanol extract contained glycosylated flavonoids with luteolin and apigenin as the main aglycones. Among them, the major compound was 7-*O*-glucosyl apigenin. Caffeoyl derivates were other phenolics identified. The essential oil obtained by steam distillation and investigated by GC/FID and GC/MS showed camphor, 1,8-cineole, and bornylacetate as the main constituents. The antioxidant capacity of three different extracts with increasing polarity and of the essential oil was evaluated by employing ABTS·+ and DPPH· radical scavenging assays. The methanolic extract was the only significantly effective sample against both synthetic radicals. All samples were also tested against Gram-positive (*Bacillus cereus*, *Enterococcus faecalis*, *Staphylococcus aureus*) and Gram-negative (*Escherichia coli*, *Proteus mirabilis*, *Pseudomonas aeruginosa*) bacterial species using the disk diffusion assay. The non-polar extracts (dichloromethane and petroleum ether) and the essential oil possessed a broad spectrum of antimicrobial activity expressed according to inhibition zone diameter (8–24 mm).

## 1. Introduction

The genus *Achillea* (Asteraceae) includes about 130 flowering and perennial species worldwide—mostly in Europe and temperate areas of Asia—but also in North America and North Africa [1,2]. *Achillea moschata* Wulfen is one of the 23 species recognized in Italy, where it grows on siliceous rocks, screes and stony pastures, along the Alps from 1800 m up to 3400 m a.s.l. [3]. It is a herb used in traditional and modern recipes for its aromatic traits and in several remedies—both in human and veterinary medicine—for various ailments, as documented in some alpine ethnobotanical studies (Table 1).

Despite its long tradition of collection and use, *A. moschata* has been poorly studied to date. Scientific reports on this plant are very few and essentially limited to germination, altitudinal adaptation and chemosystematics [12,13,14]. The oil volatile composition was characterized more than 20 years ago [15,16]. Therefore, the aim of the present work was the re-investigation of the essential oil composition and the characterization, for the first time, of the phenolic constituents in the aerial parts. In vitro antioxidant and antibacterial activities of both the essential oil and of plant extracts were also measured.

## 2. Results and Discussion

### 2.1. Characterization of the Essential Oil

The essential oil content of *A. moschata* obtained by steam distillation from the dried aerial parts of the plant was quantified as 0.81%, which was then analyzed by GC/FID and GC/MS. The constituents are reported in Table 2, wherein they are listed in ascending order of their retention indices (RIs) on a DB-5 column. A total of 40 components were identified, representing 94.36% of the oil.

Several compounds were detected belonging to different chemical classes, with monoterpenes and sesquiterpenes identified as the most abundant constituents. A series of unknown compounds were tentatively identified by comparing their MS spectra with those of known compounds.

Monoterpenes made up 81.80% of the total oil, with oxygenated monoterpenes and monoterpene hydrocarbons detected at 68.44% and 23.36%, respectively. Sesquiterpenes were found in lesser amounts than monoterpenes, and represented 7.25% of the sample. Oxigenated sesquiterpenes were the most abundant of this class of constituents, representing 5.62% of the total oil. Camphor was found to be the major constituent (27.16% of the oil,) followed by 1,8-cineole (10.69%) and bornylacetate (6.21%).

Other constituents detected in relatively high amounts were the monoterpenes: *cis-* and *trans-*thujone (4.78 and 4.82%, respectively), borneol (4.77%), *cis*-sabinene hydrate (4.28%) and camphene (3.82%). Our data were in agreement with those available from the literature [15,16] although some differences in the quantitative composition of the oil were observed, likely due to the different origin of the plant material under study.

### 2.2. Qualitative Analysis of the Methanol Extract

The phenolic compounds were characterized according to their UV, HPLC, retention times, mass spectra, and comparison with authentic standards when available. Table 3 shows the chemical composition of the methanolic extract of *A. moschata*.

TLC analyses showed several spots of different colors that were indicative of a complex metabolic profile. A typical intense fluorescence under UV-365 nm was observed immediately after spraying with NP/PEG reagent. As reported in literature [18], the fluorescence behavior is structure-dependent. In particular, flavonols such as glycosides of kaempferol and isorhamnetin give yellow-green fluorescence; flavones such as glycosides of luteolin give an orange color, while glycosides of apigenin produce yellow-green fluorescence and phenolic carboxylic acids appear as intense light blue zones. Taking into account the above reported considerations, we concluded that flavonols and phenolic acids were present in the extract flavones. TLC analyses of the two polar extracts obtained by sonification and Soxhlet (see MM) showed a superimposable profile.

These preliminary data were confirmed by ESI-MS/MS analyses. Spectra of constituents of *A. moschata* extract showed different fragmentation patterns, suggesting the presence of flavonoids and phenolic acids. In particular, flavonoids had characteristic fragmentation patterns as follows: (a) components which showed the loss of an hexose sugar from the parent ion with a base peak corresponding to the aglycone, suggesting the presence of *O*-glycosylated flavonoids; (b) components with fragment ions [(M − H) − 104]^−^; [(M − H) − 146]^−^ or [aglycone + 83]^−^, in agreement with the presence of deoxyhexose derivatives.

In addition, it was possible to confirm a pattern of fragmentation typical of caffeic acid derivatives: fragments ions at *m/z* 173 or 191 consistent with quinic acid moieties of mono-acyl or di-acyl chlorogenic acids, and fragment ions at *m/z* 179 characteristic of a caffeoyl substituent.

This information was supported by UV-data. The UV spectra showed typical behavior of flavonoids and caffeic acid derivatives. The first metabolites exhibited two major absorption bands: band I absorption in the 330–350 nm range due to the B-ring cynnamoyl system with weak absorption (*sh*) around 299 nm; band II in the range 240–280 nm due to A-ring benzoyl system for flavone derivatives while flavonols showed band II in the range 250–270 nm and band I in the range 350–390 nm.

Compounds with absorption ranges at 325.7–329.3 nm and 246.1–250.0 nm plus a diagnostic sharp shoulder at 290–300 nm were unequivocally identified as chlorogenic acids.

Two groups of major flavones were detected: a group with a band II having two absorption maxima or one maximum with a shoulder (in the range of 250–265 nm)—similar to luteolin (3′,4′,5, 7-tetrahydroxyflavone) derivatives—and another group with a maximum absorption corresponding to the apigenin (4′,5,7-trihydroxyflavone) derivatives. Moreover, the UV spectra of the extracts showed the presence of flavonols.

**Figure 1:**
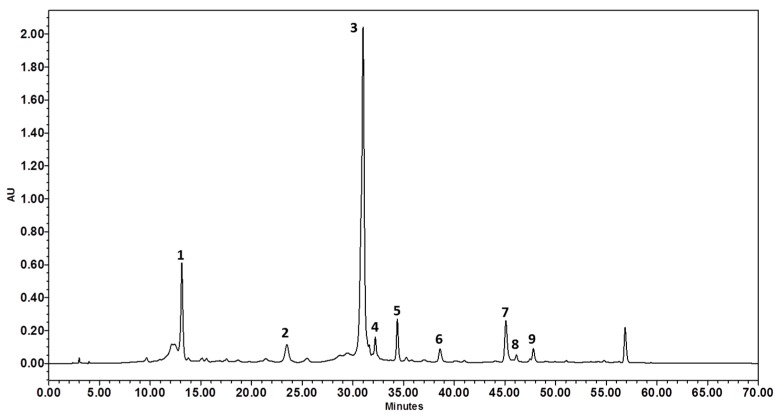
HPLC chromatogram (310 nm) of *A. moschata* methanolic extract: (1) 5-*O*-caffeoylquinic acid; (2) 7-*O*-glucosyl luteolin; (3) 7-*O*-glucosyl apigenin; (4) 4,5 dicaffeoylquinic acid; (5) 3-O-glucosyl kaempferol; (6) luteolin; (7) apigenin; (8) 3-*O*-rutinosyl isorhamnetin; (9) 3-*O*-glucosyl isorhamnetin.

The sample showed the presence of a prevalent compound (peak 3, Figure 1) with a UV spectrum typical of the apigenin derivatives. This compound was also isolated by HPLC and analyzed by ESI-MS/MS. The MS spectra showed a pseudomolecular ion at *m/z* 431 and the MS^2^ revealed the loss of one hexose unit and the appearance of the aglycone fragment at *m/z* 269 (100) [(M − H) − 162]^−^ (apigenin), due to the cleavage at the glycosidic-*O*-linkage. The metabolite was identified as 7-*O*-β-glucosyl apigenin. This result was confirmed by comparison between the retention time of the samples and the reference compound.

Peak 1 showed a UV spectra characterized by absorption at 250.0 nm, 325.7 nm and a shoulder peak at 299.0 nm. This UV spectrum is typical of phenolic compounds. The MS spectrum of this compound was characterized by a marker fragment at *m/z* 191 due to loss of a caffeic moiety (162 Da). Peak 1 was unambiguously identified as 5-*O*-caffeoylquinic acid by comparing its retention time with that of a commercial standard.

The HPLC profile of *A. moschata* presented another phenolic compound such as the peak 4 ([M − H]^−^ at *m/z* 515) corresponding to a dicaffeoylquinic acid. This compound was identified as 4,5-*O*-dicaffeoylquinic acid according to the retention time of the reference standard and the MS^2^ spectra. MS^2^ fragmentation was identical to the one previously reported by Clifford et al. [19] and Carbonara et al. [20].

Other flavones were identified in the extract, most of them associated to apigenin and luteolin derivatives based on their UV spectra (λ_max_ 330–350 nm) and MS^2^ fragmentation pattern. Luteolin-7-*O*-glucoside (peak 2) was positively identified according to the retention time, mass, and UV spectra similar to that of reference compound.

In addition, the extract was characterized by the presence of free aglycones. Peak 6 ([M − H]^−^ at *m/z* 285) isolated by HPLC and analyzed by ESI-MS/MS analysis was identified as luteolin; peak 7 ([M − H]^−^ at *m/z* 269) was identified as apigenin based on the retention time, mass, and UV spectra compared with a reference compound.

The remaining phenolic compounds in the extract corresponded to flavonols derivatives, most of them derived from kaempferol (λ_max_ around 354 nm and MS^2^ fragment at *m/z* 285) and isorhamnetin (λ_max_ 349 band I and 255 nm band II and MS^2^ fragment at *m/z* 315). The UV absorptions (Table 3) of peak 5 were consistent with a kaempferol derivative. This was supported by ESI-MS/MS analysis, which gave a pseudomolecular ion [M − H]^−^ at *m/z* 447 that fragmented giving only the ion at *m/z* 284.8 (100) [(M − H) − 162]^−^ or [aglycone (kaempferol) − H]^−^. Peak 5 was then identified as 3-*O*-β-glucosyl kaempferol.

Peak 8 at *m/z* 623 could be assigned to the isorhamnetin rutinoside based on the loss of dehoxyhexosyl and hexosyl residue (146 + 162 u) to yield the aglycone (*m/z* at 315). Based on its fragmentation and UV spectra, peak 9 was instead identified as 3-*O*-*β*-glucosyl isorhamnetin. ESI-MS/MS gave a pseudomolecular ion at *m/z* 477 [M − H]^−^ which fragmented giving a base peak at *m/z* 314.8 (100) [(M − H) − 162]^−^ and less intense fragments at *m/z* 461.9 (4) [(M − H) − 15]^−^, 356.9 (3) [(M − H) − 120]^−^ and 299.9 (23) [Aglycone – 15].

### 2.3. Quantitative Analysis of the Methanol Extract

As shown in Table 4 and Figure 1, 7-*O*-β-glucosyl apigenin was the predominant compound (33.57 µg/mg·dw) in the *A. moschata* methanolic extract followed by 5-*O*-caffeoylquinic acid (10.15 µg/mg·dw). The other components—7-*O*-β-glucosyl luteolin, 4,5-*O*-dicaffeoylquinic acid, 3-*O*-β-glucosyl kaempferol, luteolin, 3-*O*-β-glucosyl isorhamnetin, and 3-*O*-β-rutinosyl isorhamnetin—were present in comparable amounts. Finally, flavonoidic compounds were predominant compared to the caffeic acid derivatives.

The chemical composition of *A. moschata*, shown for the first time in this study, confirmed the trend that was also reported by previous investigations on chemical constituents of the genus *Achillea* identifying phenolics among the most abundant compound classes [21]. In particular, flavonoids as flavones and flavonols and their derivatives are present, in addition to hydroxycinnamic acids [22].

*A. moschata*, like other *Achillea* species, had apigenin and luteolin as free flavonoid aglycones and, in some cases, also their monoglycosides. However, many differences were present, both in relation to the type and to the amount of compounds [21,22,23,24,25]. For example, in comparison with *A. millefolium*, the unique species present in the European Pharmacopeia, *A. moschata* showed a different phenolic profile of the methanolic extract. Flavonoids were the major phenolic constituents in *A. moschata* with 7-*O*-β-glucosyl apigenin being the most abundant one, which differs from *A. millefolium* in which phenolic acids are the major metabolites. *Cis* and *trans* 3.5-*O*-dicaffeoylquinic acids predominated in *A. millefolium* [26,27], while 5-*O*-caffeoylquinic acid in *A. moschata*.

Therefore, from a phytochemical point of view, several *Achillea* species were investigated and a wide diversity was found. With regards to the phenolic compounds, the reason for such variations may be explained by the role that they play as protective agents against oxidative stress related to climatic conditions and geographical distribution. It is known that type, content and proportion of the bioactive substances synthesized by the plants may vary depending on ecological factors including the annual average precipitation, temperature, frost-free period, soil pH, and organic matter [28].

### 2.4. Antioxidant Activity

The antioxidant activity of three extracts with increasing polarity—petroleum ether (PET), dichloromethane (DCM), methanol (MeOH)—and of the essential oil (EO) from *A. moschata* was assessed by employing ABTS· + (2,2′-azino-bis(3-ethylbenzothiazoline-6-sulfonic acid) and DPPH· (2,2-diphenyl-picryl hydrazyl) radical scavenging assays. A good correlation between the two methods was observed and the results are reported in Table 5. Among the extracts, the only sample showing values useful to calculate IC_50_ was the MeOH extract. Its scavenging ability against the DPPH stable radical was higher than that of the reference compound, i.e., quercetin, in agreement with previous data from another population of *A. moschata* [29]. PET and DCM extracts had values of DPPH-scavenging lower than 50% (Table 5). Similarly, in the ABTS assay, the MeOH extract exhibited a high degree of activity, 6- and 10-fold stronger than DCM and PET extracts, respectively (Table 5). The negligible radical inhibition of the latter extracts was due to the different solvent polarity, which influences the ability to dissolve the antioxidant compounds, as widely reported [30]. The EO was able to scavenge DPPH· free radical less effectively than the MeOH extract, though with IC_50_ values still significant. Instead, the EO antiradical capacity was weak when it was tested toward ABTS· + (Table 5). There are no previous data on the antioxidant activity of *A. moschata* essential oil.

On the contrary, there are evidences to support the possible relationship between antioxidant activity and cytotoxicity. During the latest 20 years, the mechanism by which continued oxidative stress can lead to chronic inflammation—which could in turn mediate various diseases including cancer—was revealed [31]. Moreover, experimental studies (both in vitro and in vivo), demonstrated the role of plant natural compounds on growth inhibition of cancer cells [31,32,33,34]. Cytotoxic effects were also ascribed to the phenolics whose biological functions are, however, related to their structure. Compounds such as apigenin and luteolin, present in *A. moschata*, exert significant action towards specific cancer cells, while other compounds such as 7-*O*-β-glucosyl apigenin, the most abundant, were not investigated from this point of view [34,35]. Therefore, *A. moschata* could be an important cytotoxic species, also considering the strong antioxidant capacity of its methanol extract. Until now, several *Achillea* species with high antioxidant potential were tested in vitro against different tumor cell lines. They showed variable results depending on the metabolic profile of the crude extract or of the essential oil and their concentration, as well as the type of tumor cell line [22,36,37,38].

### 2.5. Antimicrobial Activity

The MeOH, DCM, and PET extracts from the aerial parts and the distilled EO from *A. moschata* were assayed for their in vitro antimicrobial properties using disk diffusion method [39]. All samples were tested against strains having a particular significance for pathogenesis, drug-resistance, and nosocomial outbreaks [40,41]. Three Gram-positive (*Bacillus cereus*, *Enterococcus faecalis*, *Staphylococcus aureus*) and three Gram-negative (*Escherichia coli*, *Proteus mirabilis*, *Pseudomonas aeruginosa*) that grow aerobically have been chosen. Moreover, we have also considered that the herb *A. moschata* is used in several remedies such as gastrointestinal disorders, skin inflammations, urinary tract inflammations, infectious diseases (see Table 1) that can be caused by these bacteria. For instance, the food-borne pathogen *B. cereus* or bacteria that are often isolated from patients with urinary tract infections—*E. faecalis*, *E. coli*, *P. mirabilis*, *P. aeruginosa*—and the multidrug-resistant *S. aureus* (MRSA) which causes invasive infections. The growth inhibition diameters of the tested samples are displayed in Table 6.

In contrast of the results of the antiradical capacity, the antimicrobial activity of the extracts was inversely proportional to their increasing polarity of the solvent used for their extraction. The MeOH extract showed a low inhibitory activity and only against *Bacillus cereus* (8 mm) and *Staphylococcus aureus* (8 mm). DCM extract was found to be active against five out of six tested bacteria (inactive against *Escherichia coli*), with inhibition diameters ranging from 8 to 15 mm. PET extract proved to be effective against all tested organisms, with inhibition diameters ranging from 8 to 15 mm.

EO demonstrated a broad spectrum of antimicrobial activity against the tested strains, in some cases greater or similar to the conventional antibiotics (erythromycin and ceftazidime). *B. cereus* was particularly susceptible, with an inhibition zone of 24 mm, followed by *S. aureus* (18 mm). The susceptibility of the other test bacteria was less pronounced, but still significant (10–13 mm). Lastly, EO showed no activity against *Enterococcus faecalis* which turned out to the most resistant among the tested microorganisms.

Disks only impregnated with extraction solvents were used as negative control and showed no inhibition of bacterial growth. The ceftazidime reference disks showed inhibition diameters ranging from 17 to 23 mm (*E. coli*, *Proteus mirabilis*, and *Pseudomonas aeruginosa*). The erythromycin reference disks showed inhibition diameters ranging from 16 to 20 mm (*B. cereus* and *S*. *aureus*), while no inhibitory effect was observed against *E. faecalis*.

No previous data about the antimicrobial activity of *A. moschata* have been reported until now. However, some studies were carried out on other *Achillea* species. Their polar extracts, in agreement with our results, mostly showed weak or no inhibitory effect against the test microrganisms [42,43,44]. These findings were also in accordance with those of Sökmen and co-authors [45] who observed no antimicrobial activity in the water soluble part of methanol extract from *A. biebersteinii*, while water-insoluble (CHCl_3_) part characterized by less polar compounds was found to have moderate activity against some tested bacteria. The only sample which was strongly active derived from a plant extract obtained by mixing ether, hexane and MeOH (1:1:1) [46]. The antibacterial properties of EO were probably due to their high content of camphor and 1,8-cineol, whose noticeable antimicrobial potential is known [21] as well as that of borneol [47].

A more detailed comparison of the results is difficult due to the fact that the same type of extract and essential oil may vary in efficacy if obtained from the same species collected in different locations. In fact, soil, altitude, and other environmental factors can influence the type and the levels of antimicrobials synthesized by the plant [48].

## 3. Materials and Methods

### 3.1. General

Standards of flavonoids 7-*O*-β-glucosyl luteolin, apigenin, and 7-*O*-β-glucosyl apigenin were purchased from Extrasynthese (Genay, France). 5-*O*-caffeoylquinic acid, HPLC grade water, and acetonitrile were purchased from Sigma-Aldrich (Milan, Italy) and 3,4-*O*-dicaffeoylquinic acid, 4,5-*O*-dicaffeoylquinic acid and 3,5-*O*-dicaffeoylquinic acid were obtained from Phytolab (Vestenbergsgreuth, Germany).

Diethyl ether and a series of authentic reference compounds used for identification of the essential oil components were also purchased from Sigma-Aldrich (Milan, Italy).

### 3.2. Plant Material

The aerial parts of *A. moschata* were collected at the blossom period during the summer 2013, at 2400 m above sea level in Valle dei Forni (Sondrio province, Italy), on the Rhaetian Alps. A voucher specimen (No. AMSHT 103) was deposited at the Department of Agricultural and Environmental Sciences of the Milan State University (Milan, Italy), after its identification according to Flora d’Italia [3].

### 3.3. Isolation of Essential Oil

The air-dried aerial parts (24 g) of the sample were subjected to steam-distillation for 1 h in a Clevenger-type apparatus. The distillate was saturated with NaCl, extracted with freshly distilled Et_2_O (3 × 100 mL), dried over anhydrous Na_2_SO_4_ and concentrated with a rotary evaporator at 30 °C to give a pale-blue yellow oil with a yield of 0.81%. The oil was diluted with Et_2_O and then used for gaschromatographic analyses.

### 3.4. Analysis of the Essential Oil

#### 3.4.1. Gas Chromatography-Flame Ionization Detector (GC/FID)

GC/FID analyses were carried out using a Perkin Elmer model 8500 GC equipped with a 30 m × 0.32 mm i.d. Elite-5MS capillary column (0.32 μm film thickness). Samples (0.5 μL) were injected in the “split” mode (1:30) with a column temperature programme of 40 °C for 5 min, then increased to 260 °C at 4 °C/min and finally held at this temperature for 10 min. The injector and the detector were set at 250 and 300 °C, respectively; the carrier gas was He with a head pressure of 12.0 psi.

#### 3.4.2. Gas Chromatography-Mass Spectrometry (GC/MS)

GC/MS analyses were carried out using a Perkin Elmer Clarus 500 GC equipped with a Clarus 500 mass spectrometer using the same capillary column and chromatographic conditions as was used for GC/FID analysis. The mass spectra were acquired over 40–500 amu range at 1 scan/s with ionizing electron energy of 70 eV and the ion source set at 200 °C. The transfer line was set at 280 °C, while the carrier gas was He at 1.0 mL/min.

### 3.5. Identification and Quantitation of the Essential Oil Components

The identification of the volatile oil components was performed by their Retention Indices (marked as RI in Table 2) and their mass spectra, by comparison with a NIST database mass spectral library [49], as well as with literature data [17,50].

Authentic reference compounds purchased from Sigma-Aldrich were also used. Retention indices were calculated using a *n*-alkane series (C_6_–C_32_) under the same GC conditions as for the samples. The relative amounts of individual components of the essential oil were expressed as percent peak area relative to total peak area from the GC/FID analyses of the whole extracts.

### 3.6. Extraction and Isolation

Hydroalcholic extract was obtained from dry aerial parts of *A. moschata* plants. The sample (4 g) was extracted twice by sonicator with methanol 80% (150 mL × 2) for 30 min, and subsequently filtered through a filter paper. The combined hydroalcholic extracts were evaporated at 30 °C (rotary evaporator) to dryness. The dry extract was re-dissolved in methanol (final concentration 30 mg/mL) for phenolic compound identification and quantification. In addition, the sample was extracted with Soxhlet in methanol (9 g × 3 h). To obtain non-polar extracts, aerial parts were also defatted with petroleum ether and successively extracted with dichloromethane (Soxhlet apparatus), then concentrated under reduced pressure (rotary evaporator) to dryness and stored at 4 °C until analyses.

#### 3.6.1. TLC

The preliminary screening of the obtained polar extracts (final concentration 30 mg/mL) was performed with TLC (precoated silica gel 60 F254 aluminium plates, Merck, Milan, Italy) eluted with ethylacetate/formic acid/acetic acid/water (100:11:11:27 *v*/*v*). Visualization of compounds was obtained with Natural Product-Polyethylenglycol Reagent (NP/PEG, Sigma, Milan, Italy). After spraying, TLC plates were observed under UV-366 nm light.

#### 3.6.2. HPLC

The HPLC analysis was performed on a Waters HPLC 600 Liquid Chromatograph (Milan, Italy) instrument equipped with a photo diode-array detector, DAD 2828 Waters. Data were processed with Empower™ 2 Waters Software. The analyses were running on a Gemini C18 (Phenomenex) column (250 × 4.6 mm i.d.; 5 µm particle size). The mobile phase was water containing 0.1% (*v*/*v*) formic acid (A) and acetonitrile with 0.1% (*v*/*v*) of formic acid (B). The linear gradient started from 10% B and reached to 60% B in 60 min. The flow rate was 1 mL/min. UV spectra of the extract were conventionally recorded at 210, 270, 310 and 350 nm. All analyses were run in triplicate. An aliquot of 20 μL of each polar extract was injected for each run. For quantitative analysis, 5-level calibration curve was obtained by injection of known concentrations (10–250 µg/mL) of different standards compounds: luteolin-6-*C*-glucoside (y = 44625x + 119464; *R*^2^ = 0.9992) and 5-*O*-caffeoylquinic acid (y = 32345x – 58693; *R*^2^ = 0.9999).

#### 3.6.3. ESI-MS/MS

Flow injection MS analyses were performed on a 1100 Series Agilent LC/MSD Trap-System VL. An Agilent Chemstation (LC/MSD TrapSoftware 4.1, Agilent Technologies: Santa Clara, CA, USA, 2002) was used for the acquisition and processing of the data. All analyses were carried out using a ESI ion source in the negative mode with the following settings: capillary voltage, 4000 V; nebulizer gas (N2), 15 psi; drying gas (N2), 350 °C, 5 L/min. Full scan spectra were acquired over the range of 100–2200 *m/z* with a scan time of 13,000 *m/z*/s. Automated ESI-MS/MS was performed by isolating the base peaks (molecular ions) using an isolation width of 4.0 *m/z*, threshold set at 100 and ion charge control on, with max acquire time set at 300 ms. Different collision energies were conventionally used (1.0, 10 and 30 V) for MS/MS fragmentation. Polar samples were dissolved in MeOH at the concentration of 20–30 ppm and injected at a flow rate of 10 µL/min (KD Scientific Syringe Pump).

### 3.7. Antiradical Activity

#### 3.7.1. DPPH**^.^** Radical-Scavenging Assay

The 2,2-diphenyl-picryl hydrazyl (DPPH**^.^**) radical-scavenging capacity was performed following Iriti et al. [51], with some modifications. In brief, aliquots of each sample, at five different concentrations (from 1–100 μM), were added to 0.07 mM MeOH solution of DPPH· free radical reaching a final volume of 2 mL. After a reaction time of 30 min in the dark at room temperature, the decrease in absorbance was measured at 517 nm. The IC_50_ was calculated with Prism^®^4 (GraphPad Software Inc., La Jolla, CA, USA).

#### 3.7.2. ABTS·^+^ Radical-Scavenging Assay

The 2,2′-azino-bis(3-ethylbenzothiazoline-6-sulfonic acid) (ABTS·^+^) radical cation-scavenging capacity was determined according to Iriti et al. [51]. The ABTS·^+^ radical cation was produced by reacting 7 mM ABTS with 2.45 mM potassium persulfate (final concentration) and maintaining the mixture in the dark at room temperature for at least 6 h before use. The ABTS·^+^ solution was diluted with ethanol to an absorbance of 0.7 (±0.02) at 734 nm and equilibrated at 30 °C. Ten μL of each sample, ethanol (negative control) and standard solution of the synthetic antioxidant 6-hydroxy-2,5,7,8-tetramethychroman-2-carboxylic acid (Trolox, positive control) were mixed for 30 s with one mL of diluted ABTS·^+^ solution. Their absorbance was read at 734 nm, at room temperature, 50 s after the initial mixing. The results are expressed as Trolox equivalent antioxidant capacity (TEAC, μmol·eq·Trolox mL).

### 3.8. Antimicrobial Activity

The disk diffusion method was used according to the standard protocol of Bauer et al. [39] to assess antibacterial activities both of the investigated plant extracts and the essential oil. The following reference strains were included in the study: three Gram-positive (*Bacillus cereus* ATCC 999091, *Enterococcus faecalis* ATCC 29212, and *Staphylococcus aureus* ATCC 25923) and three Gram-negative (*Escherichia coli* ATCC 35218, *Proteus mirabilis* ATCC 12453, and *Pseudomonas aeruginosa* ATCC 27853). All organisms were maintained in brain-heart infusion (BHI medium Oxoid, Milan, Italy) containing 30% (*v*/*v*) glycerol at −80 °C. Before testing, the suspensions were transferred in BHI and overnight bacterial cultures were adjusted to 0.5 McFarland standards to achieve an inoculum of approximately 106 CFU/mL, and individually uniformly spread on Muller Hinton agar (MH, Oxoid, Milano) plates using a sterile swab. Each plate was dried for 15 min and then used for the sensitivity test. Sterile paper disks, 6 mm in diameter (Oxoid, Milano), which had previously been impregnated with 10 μL of each plant extract (MeOH, DCM, PET) and of the essential oil were dried, and then aseptically placed onto the surface of the inoculated media. Disks impregnated with extraction solvents were used as negative controls and disks with ceftazidime (30 μg) and erythromycin (5 μg) as references for Gram-negative and Gram-positive bacteria, respectively. After incubation at 37 °C for 18 h, plates were examined and the inhibition zones around the disks were measured (diameters in mm). Tests were repeated three times for each extract/microorganism combination.

## 4. Conclusions

This study reported, for the first time to the best of our knowledge, the characterization of the phenolic content and antibacterial activity of *A. moschata*, in addition to showing the high antiradical ability of the methanolic extract. Moreover, we have integrated the data available about the essential oil composition and we have presented the oil’s early results related to its biological activity, which was never investigated before.

Our results, although preliminary, provide evidence to support some of the documented ethnobotanical uses of *A. moschata* that suggest its aerial parts can be considered a good source of bioactive compounds, potentially exploitable in the pharmaceutical field. To this end, future studies will focus on the identification of the antimicrobials in the most effective extracts, in addition to investigating other biological activities.

## Figures and Tables

**Table 1:** Traditional uses of *A. moschata* as documented in the ethnobotanical reports.

Use Category	Ailment	Preparation and Administration	References
Medicine	Abdominal bloating, colic, cold, cough, dysmenorrhea, earache, fever, flatulence, gastrointestinal disorders, gout, headache, hypertension, insomnia, menopausal disorders, neuralgia, oliguria, poor digestion, skin inflammations, urinary tract inflammations, vaginitis	Infusion (to drink, to make compresses); Liqueur; Ointment (to rub)	[4,5,6,7,8,9,10,11]
Veterinary medicine	Digestion and skin inflammations (cows and calves)	Infusion (to drink, to make compresses)	[8,9,10,11]
Food	Flavouring of typical dishes, soups, desserts, *grappa*, liqueurs, spicing cheese		[10,11]

**Table 2:** Percentage composition of essential oil from *A. moschata*.

#	Compound	RI ^a^	RI ^b^	%
1	santolina triene	902	906	0.51
2	tricyclene	916	921	0.24
3	artemisia triene	923	923	0.58
4	α-thujene	928	924	1.38
5	camphene	943	946	3.82
6	sabinene	968	969	1.83
7	β-pinene	971	974	0.84
8	1,8-dehydrocineole	986	988	0.03
9	myrcene	992	988	2.23
10	α-terpinene	1013	1013	0.10
11	p-cymene	1021	1020	1.30
12	1,8-cineole	1028	1026	10.69
13	γ-terpinene	1055	1054	0.53
14	*cis*-sabinene hydrate	1067	1065	4.28
15	*trans*-sabinene hydrate	1097	1098	0.88
16	*cis*-tujone	1102	1101	4.78
17	isovaleric acid 2-methylbutyl ester	1109	1103	0.20
18	*trans*-tujone	1114	1112	4.82
19	chrysantenone	1117	1124	1.43
20	camphor	1142	1141	27.16
21	pinocarvone	1157	1160	0.31
22	borneol	1167	1165	4.77
23	terpinen-4-ol	1176	1177	1.26
24	*α*-terpineol	1191	1186	1.43
25	*trans*-piperitol	1205	1207	0.02
26	bornylacetate	1280	1287	6.21
27	thymol	1289	1289	0.26
28	carvacrol	1297	1298	0.11
29	β-caryophyllene	1412	1419	1.38
30	sesquicineole	1508	1515	1.31
31	sesquiterpene hydrocarbon C_15_H_24_ MW = 204	1558	-	0.25
32	oxigenated sesquiterpene C_15_H_22_O MW = 218	1565	-	0.37
33	oxigenated sesquiterpene C_15_H_26_O MW = 222	1569	-	0.15
34	caryophyllene oxide	1573	1582	1.00
35	oxigenated sesquiterpene C_15_H_24_O MW = 220	1607	-	0.40
36	oxigenated sesquiterpene C_15_H_26_O MW = 222	1625	-	0.73
37	oxigenated sesquiterpene C_15_H_24_O MW = 220	1629	-	1.01
38	oxigenated sesquiterpene C_15_H_26_O MW = 222	1646	-	0.65
39	unidentified C_15_H_26_O_2_ MW = 238	1679	-	0.65
40	unidentified C_15_H_26_O_2_ MW = 238	1743	-	4.46
	Total monoterpenes			81.80
	Total sesquiterpenes			7.25
	Others			5.31
	Total			94.36

RI ^a^: Retention Indices from literature data [17]. RI ^b^: Retention Indices calculated by GC/MS using n-alkane series (from C_6_ to C_32_) under the same analytical conditions as for the samples.

**Table 3:** Summary of phenolic compounds identified in the methanolic extract from *A. moschata*.

R_t_ (min)	Name	UV (λ_max_, nm)	[M − H]^−^ (*m/z*)	ESI-MS/MS (%)
13.12	5-*O*-Caffeoylquinic acid	250; 299.0 (*sh*); 325.7	353	190.7 (100) [(M − H) − 162]^−^; 178.7 (27) [(M − H) − 174]^−^
23.46	7-*O*-β-Glucosyl luteolin	255.5; 267.4 (*sh*); 349.7	447	284.8 (100) [(M − H) − 162]^−^, [Aglycone − H]^−^
31.00	7-*O*-β-Glucosyl apigenin	266.2; 334.1	431	310.8 (3) [(M − H) − 120]^−^; 268.8 (100) [(M − H) − 162]^−^, [Aglycone − H]^−^
32.26	4,5-*O*-Dicaffeoylquinic acid	246.0; 298.8; 329.3	515	352.9 (100) [(M − H) − 162]^−^; 190.7 (13) [(M − H) − 324]^−^; 178.7 (8) [(M − H) − 162 − 174]^−^; 172.7 (4) [(M − H) − 162 − 162 − 18]^−^
34.38	3-*O*-β-Glucosyl-kaempferol	266.2; 354.1	447	284.8 (100) [(M − H) − 162]^−^, [Aglycone − H]^−^
38.60	Luteolin	250.0; 254.3; 299.0 (*sh*); 347.7	285	356.7 (16) [(M − H) − 74]^−^; 326.8 (100) [(M − H) − 104]^−^; 284.7 (1) [(M − H) − 146]^−^, [Aglycone − H]^−^
45.11	Apigenin	267.4; 299.7 (*sh*); 336.5	269	310.8 (3) [(M − H) − 120]^−^; 268.8 (100) [(M − H) − 162]^−^, [Aglycone − H]^−^
45.89	3-*O*-β-Rutinosyl-isorhamnetin	258.4; 276.1 (*sh*); 349.5	623	314.8 (100) [(M − H) − 162 − 146]^−^, [Aglycone − H]^−^; 299.8 (29) [(Aglycone − H) − 15]^−^
47.79	3-*O-*β-Glucosyl-isorhamnetin	255.5; 275.7 (*sh*); 349.7	477	314.8 (100) [(M − H) − 162]^−^, [Aglycone − H]^−^; 299.9 (23) [(Aglycone − H) − 15]^−^

**Table 4:** Quantification results for phenolic compounds in the methanolic extract from *A. moschata*.

Peak No	Name	µg/mg·dw
1	5-*O*-caffeoylquinic acid	10.15 ± 0.19
2	7-*O*-glucosyl luteolin	2.20 ± 0.06
3	7-*O*-β-glucosyl apigenin	33.57 ± 0.93
4	4,5-dicaffeoylquinic acid	2.53 ± 0.18
5	3*-O*-β-glucosyl-kaempferol	3.43 ± 0.05
6	Luteolin	1.10 ± 0.25
7	Apigenin	3.43 ± 0.02
8	3-*O*-β-Rutinosyl-isorhamnetin	1.27 ± 0.10
9	3-*O*-β-Glucosyl-isorhamnetin	3.29 ± 0.04
Total Caffeic compounds		12.68
Total Flavonoidic compounds		48.29

**Table 5:** In vitro antioxidant activity of the extracts and essential oil from aerial parts of *A. moschata* measured by DPPH· and ABTS·+ radical scavenging assays.

Samples *	DPPH (IC_50_) μM	ABTS (μmol·eq·Trolox/g)
MeOH	3.18 ± 0.09	502.44 ± 0.01
DCM	n/a	88.47 ± 0.01
PET	n/a	48.39 ± 0.01
EO	47.70 ± 0.78	5.88 ± 0.01
Quercetin	4.39 ± 0.12	-

* MeOH, methanol extract; DCM, dichloromethane extract; PET, petroleum ether extract; EO, essential oil. Tests were performed in triplicate; means ± SD.

**Table 6:** Growth inhibition diameters (mm) against bacterial strains recorded for MeOH *, DCM, PET extracts and EO from the aerial parts of *A. moschata*.

Bacteria	MeOH	DCM	PET	EO
*B. cereus*	8	15	10	24
*E. faecalis*	-	11	11	-
*S. aureus*	8	12	15	18
*E. coli*	-	-	13	11
*P. mirabilis*	-	10	15	13
*P. aeruginosa*	-	8	8	10

* MeOH, methanol extract; DCM, dichloromethane extract; PET, petroleum ether extract; EO, essential oil. The values represent the mean of inhibition zone diameters (including disk diameter of 6 mm). For all values the standard deviation is <1 mm.
